# Deciphering the regulatory mechanisms of potato cold-induced sweetening via integrated time-course transcriptome and metabolome analysis

**DOI:** 10.3389/fpls.2025.1551265

**Published:** 2025-04-15

**Authors:** Guangcan Cui, Tingting Zhou, Zhongliang Liu, Ting Wang, Qingguo Wang, Tengfei Liu

**Affiliations:** ^1^ College of Food Science and Engineering, Shandong Agricultural University, Tai’an, Shandong, China; ^2^ State Key Laboratory of Wheat Breeding, College of Agronomy, Shandong Agricultural University, Tai’an, China; ^3^ Department of Agronomy, Kansas State University, Manhattan, KS, United States; ^4^ Tai’an Academy of Agricultural Sciences, Tai’an, China

**Keywords:** potato, cold-induced sweetening, processing quality, transcriptome, co-expression analysis

## Abstract

Cold-induced sweetening (CIS) in potatoes is a significant challenge affecting the quality and marketability of processed potato products. This study aims to decipher the regulatory mechanisms underlying CIS through integrated time-course transcriptome and metabolome analysis. Freshly harvested potato tubers (*Solanum tuberosum* L. cv. ‘Netherlands No. 15’) were stored at 4°C and 20°C for various durations. RNA sequencing and quantitative real-time PCR were employed to analyze gene expression changes, while metabolomic profiling was conducted using UHPLC-MS/MS. Our results reveal significant alterations in carbohydrate metabolism, with a marked increase in reducing sugars during cold storage. Critical enzymes involved in starch degradation, such as β-amylases (*StBAM1*, *StBAM9*), and sucrose metabolic genes (*StUGPase2*, *StVInv*) were upregulated, while starch synthesis genes were downregulated. Co-expression analysis identified potential transcription factors, including *StHSFA2* and *StAPRR2*, which may regulate these metabolic pathways. Functional assays demonstrated that StHSFA2 significantly activates the promoters of *StBAM1* and *StUGPase2*, while StAPRR2 shows specific activation of the *StVInv* enhancer. These findings suggest distinct but complementary roles for StHSFA2 and StAPRR2 in regulating starch degradation and sucrose metabolism during CIS. This comprehensive analysis provides insights into the molecular mechanisms of CIS and identifies potential targets for genetic manipulation to mitigate its effects, thereby enhancing the storage quality and processing performance of potatoes.

## 1 Introduction

Cold-induced sweetening (CIS) in potatoes is a significant challenge affecting the quality and marketability of processed potato products. When potatoes are stored at low temperatures (approximately 4°C), they undergo a physiological process that leads to the accumulation of reducing sugars (primarily glucose and fructose), which negatively impacts their processing quality (Sowokinos, 2001). Cold storage effectively suppresses sprouting, minimizes water loss, and curbs the proliferation of diseases in tubers. However, exposure to cold triggers the degradation of starch and the accumulation of reducing sugars (RS, mainly glucose and fructose) in tubers (Sowokinos, 2001).

When these sugar-rich potatoes undergo high-temperature processing for products like chips or fries, the RS react with asparagine, undergoing the Maillard reaction and consequently producing the toxic compound acrylamide (Shepherd et al., 2010). Beyond the health concerns, this also imparts an unwelcome dark tint to the final products (Bhaskar et al., 2010). Statistically, approximately 20% of tubers designated for processing in the United States are discarded annually due to excessive RS levels (Wiberley-Bradford et al., 2014). Lowering the RS concentration in the raw material effectively reduces acrylamide formation in potato-derived products (Bhaskar et al., 2010). Contemporary consumers exhibit a greater willingness to purchase potato products with health-related improvements (such as lower acrylamide) (Lukasiewicz et al., 2024). Thus, tackling and mitigating CIS becomes imperative to capitalize on cold storage for the potato industry genuinely.

Researchers have diligently investigated the mechanisms underlying CIS, making notable advancements. Studies indicate that cold storage stimulates starch degradation pathways, leading to sucrose resynthesis and subsequent sucrose breakdown, which are critical processes in CIS (Sowokinos, 2001). The roles of key enzymes and critical genes involved in these pathways during the CIS process have also been elucidated. In the starch degradation pathway, suppression of *α-glucan, water dikinase* (*GWD*) can mitigate the effects of CIS (Lorberth et al., 1998); three amylase genes, *StAmy23*, *StBAM1*, and *StBAM9*, influence CIS differently (Hou et al., 2017). Meanwhile, the amylase inhibitor SbAI impacts CIS by moderating amylase activity (Zhang et al., 2014). Silencing *UDP-glucose pyrophosphorylase* (*UGPase*), which catalyzes the first reversible and committed step in sucrose synthesis by forming UDP-glucose, attenuates CIS (Jaiswal et al., 2023). Tonoplast sugar transport (TST) 1 mediates the sucrose uptake from the cytosol to the vacuole, and inhibition of *TST1* can ameliorate CIS (Liu et al., 2023b). In the vacuole, sucrose is further hydrolyzed by invertase to produce RS, representing the direct step in CIS. Restraining the expression of the vacuolar invertase gene *StVInv*/*Pain-1* or overexpression of its inhibitor both effectively inhibit CIS (Bhaskar et al., 2010; Liu et al., 2011, 2013; Mckenzie et al., 2013). Additionally, the α and β subunits of the Sucrose Nonfermenting1-Related Protein Kinase (SnRK1) establish a protein complex in conjunction with the invertase and its inhibitor, finely tuning the acid invertase activity and thus holding pivotal roles in CIS (Lin et al., 2015). Comparative transcriptome and co-expression analyses of potato varieties with varying CIS resistance identified multiple critical TFs potentially regulating CIS resistance (Liu et al., 2021). Subsequent functional analyses demonstrated that StRAP2.3 and StTINY3 enhance CIS resistance. StRAP2.3 functions by directly activating StInvInh2, while StTINY3 operates by coordinating the processes of starch resynthesis and sucrose hydrolysis (Shi et al., 2021, 2022).

Despite significant progress in understanding CIS, the regulatory mechanisms by which potato tubers sense and respond to cold stimuli, leading to starch-sugar interconversion, remain largely unexplored. Specifically, the transcription factors (TFs) that control the expression of genes involved in CIS have not been comprehensively identified and characterized.

Given CIS’s economic and quality implications in potato storage and processing, this study aims to deepen the understanding of the molecular mechanisms underlying CIS, mainly focusing on the transcriptional and metabolic changes that occur during cold storage. The study seeks to identify critical TFs and metabolic pathways that regulate starch-sugar interconversion and contribute to CIS by employing transcriptome-based co-expression analysis and metabolomics. Additionally, the study will explore the roles of specific *TF*s in regulating these processes. Ultimately, this research aims to provide new insights that could inform the development of strategies to mitigate CIS, thereby enhancing the quality and shelf life of stored potatoes and supporting the potato processing industry’s need for consistent, high-quality raw materials.

## 2 Materials and methods

### 2.1 Tuber treatments, potato chipping analysis, and sugar and acrylamide determination

Potato (*Solanum tuberosum* L., cv. ‘Netherlands No. 15’) and soybean oil (Luhua Rich Fragrance Soybean Oil, Shandong Luhua Group Co., Ltd.) were purchased in April 2023 from a local market in Tai’an City, Shandong Province, China. Tubers devoid of mechanical damage, pest infestation, and diseases and uniform size were chosen for post-harvest storage experiments. The storage protocol entailed maintaining the tubers under dark conditions at 4°C and 20°C. Specifically, freshly harvested potatoes were stored at 4°C for intervals of 0, 1, 3, 5, 10, and 15 days, and at 20°C for 5, 10, and 15 days. For each storage condition, the experimental groups consisted of at least six tubers.

Potato chipping analysis and sugar and acrylamide determination were conducted as in our previous report (Liu et al., 2023b).

### 2.2 Transcriptomic analysis

Total RNA was extracted from potato tuber tissue using the Plant Total RNA Kit (ZOMANBIO, Beijing, China) and assessed for quality with a NanoDrop 1000 Spectrophotometer (Thermo Scientific, Waltham, MA, USA). First-strand cDNA was synthesized using the HifiScript cDNA Synthesis Kit (Cowin Biosciences, Beijing, China). For RNA sequencing, libraries were constructed from 2 μg RNA per sample and sequenced on an Illumina platform (Smartgenomics, Tianjin, China), with 21 libraries representing three biological replicates per time point. Clean reads were processed as per Liu et al. (2023a), and transcript quantification was performed using Salmon with the DM1-3 v6.1 reference (http://spuddb.uga.edu/dm_v6_1_download.shtml) (Patro et al., 2017). Differentially expressed genes (DEGs) were identified using DESeq2 (FDR < 0.01, |log2FC| ≥ 1) (Love et al., 2014). Transcripts Per Million (TPM) values facilitated cross-sample comparisons, with principal component analysis (PCA) of the top 5000 TPM-ranked genes and DEG overlaps visualized via UpSetR (Conway et al., 2017).

For quantitative real-time PCR (qRT-PCR), the potato Ef1α gene (XM_006343394) was used as the internal reference. Reactions were run on a Bio-Rad CFX96 system with the Ultra SYBR Mixture Kit (Cowin Biosciences), and relative expression was calculated per Liu et al. (2019). Primers are listed in **Supplementary Table S1**.

### 2.3 Metabolite extraction

The samples used for metabolite profiling were derived from the same set of biological materials as previously mentioned for RNA-seq analysis, ensuring direct comparability between metabolomic and transcriptomic data. The tuber samples are lyophilized and precisely weighed (25 mg) into Eppendorf tubes, followed by the addition of a homogenization bead and 1000 μL of extraction solution, which consists of a mixture of methanol, acetonitrile, and water (2:2:1, V/V) and contains a blend of isotope-labeled internal standards. The samples are then vortexed for 30 seconds to achieve thorough mixing. Next, the samples are transferred to a homogenizer and subjected to homogenization at 35 Hz for 4 minutes, followed by placement in an ice-water bath and sonication for 5 minutes, with this step being repeated three times to enhance the extraction efficiency. Subsequently, the samples are incubated at -40°C for 1 hour to promote protein precipitation and improve metabolite recovery. After incubation, the samples are centrifuged at 4°C and 12000 rpm (centrifugal force 13800 ×g, radius 8.6 cm) for 15 minutes, and the supernatant is collected. The supernatant is then subjected to a second round of centrifugation under the same conditions to remove any remaining particulate matter. Finally, the resulting supernatant is transferred into sample vials for subsequent instrumental analysis.

### 2.4 LC-MS/MS analysis and data preprocessing

For the analysis of polar metabolites, a UHPLC system (Vanquish, Thermo Fisher Scientific) equipped with a Waters ACQUITY UPLC BEH Amide column (2.1 mm × 100 mm, 1.7 μm) was utilized, connected to an Orbitrap Exploris 120 mass spectrometer (Orbitrap MS, Thermo Fisher Scientific). The mobile phase included 25 mmol/L ammonium acetate and 25 mmol/L ammonium hydroxide in water (pH = 9.75) as phase A and acetonitrile as phase B. The auto-sampler was maintained at 4°C, and the injection volume was set at 2 μL.

For the analysis of non-polar metabolites, the same UHPLC system (Vanquish, Thermo Fisher Scientific) was used with a Phenomenex Kinetex C18 column (2.1 mm × 100 mm, 2.6 μm), also coupled to the Orbitrap Exploris 120 mass spectrometer. The mobile phases were: A - 0.01% acetic acid in water and B - a mixture of isopropanol and acetonitrile (1:1, v/v). The auto-sampler was set at 4°C, with an injection volume of 2 μL.

The Orbitrap Exploris 120 mass spectrometer was operated in information-dependent acquisition (IDA) mode controlled by the Xcalibur software (Vanquish, Thermo Fisher Scientific). This mode continuously evaluates the full scan MS spectrum. The ESI source conditions were as follows: sheath gas flow rate at 50 Arb, auxiliary gas flow rate at 15 Arb, capillary temperature at 320°C, full MS resolution at 60000, MS/MS resolution at 15000, collision energy settings at SNCE 20/30/40, and spray voltage at 3.8 kV (positive mode) or -3.4 kV (negative mode).

The raw data were converted to the mzXML format using ProteoWizard. The converted data were then processed with an in-house program developed in R, utilizing the XCMS package for peak detection, extraction, alignment, and integration. Metabolite identification was performed using the R package and the BiotreeDB (V3.0).

### 2.5 KEGG and GO enrichment analysis

KEGG pathway enrichment analysis was conducted using TBtools (Chen et al., 2020), applying a hypergeometric test to identify significantly enriched pathways with an adjusted *P*-value threshold set at < 0.05. For GO enrichment analysis, the BiNGO plugin within CYTOSCAPE (Maere et al., 2005) was utilized, employing a stringent significance criterion based on the Benjamini & Hochberg False Discovery Rate (FDR), set to be below 0.01.

### 2.6 Clustering and heatmap analysis

For clustering analysis, each treatment’s Transcripts Per Million (TPM) values were first normalized, converting each gene’s expression profile to have a mean of zero and a standard deviation of one. This standardized dataset was then subjected to fuzzy c-means (FCM) clustering, implemented through the Mfuzz package (Kumar and Futschik, 2007). FCM clustering assigns a membership score between 0 and 1 to each gene, reflecting the degree to which the gene is representative of a particular cluster. This method relies on Euclidean distance as the metric for similarity assessment. Two key parameters were optimized through iterative processes: ‘c’, representing the number of clusters, was set to 6, and ‘m’, which controls the influence of noise in the clustering process, was determined to be 1.75. Visualization and heatmap generation were performed using the TBtools, providing a clear graphical representation of gene expression patterns across different conditions.

### 2.7 Subcellular localization analysis of candidate transcription factors

To elucidate the subcellular localization patterns of potato HSF and APRR-B proteins, we performed a transient expression system in *Nicotiana benthamiana*. The complete coding regions of each gene, including the stop codon, were amplified from potato cDNA using specific primers (**Supplementary Table S1**). The resultant amplicons were subsequently inserted into the pH7LIC-N-eGFP vector using Exnase II (Vazyme, Nanjing, China), generating constructs that express N-terminal GFP-tagged fusion proteins *in vivo*. These recombinant plasmids were introduced into *Agrobacterium tumefaciens* strain GV3101 for transient expression in *N. benthamiana* leaf epidermal cells via agroinfiltration. Sixty hours post-infiltration, the subcellular distribution of the GFP-HSF and GFP-APRR-B fusion proteins was visualized using a Leica TCS-SPE confocal laser scanning microscope (Leica, Wetzlar, Germany).

### 2.8 Evaluating the transcriptional regulation of *StBAM1* and *StVInv* by candidate TFs

To investigate the potential regulatory effects of StHSFA2 and StAPRR2 on the expression of *StBAM1* and *StVInv*, we performed dual-luciferase reporter assays in *Nicotiana benthamiana*. The reporter vector for *StBAM1*, designated as *StBAM1Promoter*-*LUC* and obtained in our previous study (Liu et al., 2023b), was constructed using a 1,426-bp genomic fragment that spans from -1,404 bp to +34 bp relative to the transcription start site of *StBAM1* to insert upstream of the firefly *luciferase* (*LUC*) reporter gene to drive its expression. For *StVInv*, a recent study has revealed that the induction of its expression during cold storage is driven by an approximately 200-bp enhancer, *VInvIn2En*, located within the second intron (Zhu et al., 2024). Therefore, we cloned a genomic fragment containing this regulatory sequence and inserted it upstream of the luciferase (LUC) reporter gene to generate the *VInvIn2En-LUC* reporter vector. The primer sequences used for gene amplification and vector construction are listed in **Supplementary Table S1**.

GFP-StHSFA2 and GFP-StAPRR2 served as effector constructs in this study. Agrobacterium strains harboring the reporter and effector plasmids were co-infiltrated into *N. benthamiana* leaves, with three biological replicates per combination. Seventy-two hours after agroinfiltration, the LUC and Renilla luciferase (REN) activities in the infected leaf samples were quantified using the Dual-Luciferase Reporter Assay System (Promega, Madison, WI, USA). The LUC to REN activity ratio was calculated to measure each TF protein’s transcriptional activation of the *StBAM1* promoter or the *StVInv* enhancer.

## 3 Results

### 3.1 Post-harvest storage impact on potato sugar levels, processing quality, and acrylamide formation

**Figure 1:**
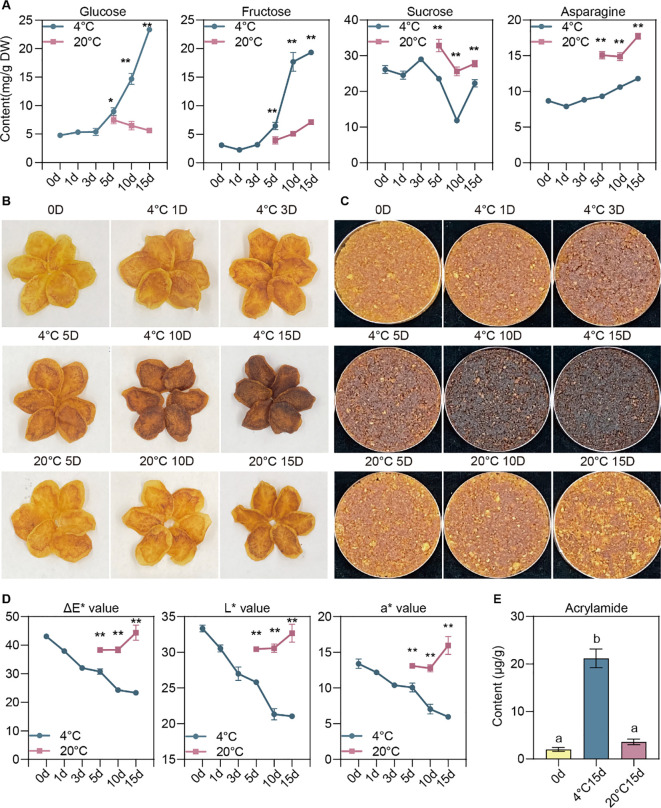
Effects of post-harvest storage on potato processing quality. **(A)** Dynamic alterations in glucose, fructose, sucrose, and asparagine levels in potato tubers subjected to varied storage conditions. **(B)** The physical appearance of potato chips processed from tubers stored under different conditions. **(C)** Phenotypes of the potato chip-derived powders. **(D)** Statistics of ΔE^*^, L^*^, and a^*^ values of potato chip-derived powders. **(E)** Acrylamide levels in potato chips produced from tubers stored for durations of 0 days, 15 days at 20°C, and 15 days at 4°C. The data are shown as means ± standard deviation (SD) for three replicates (n=3). The symbols * and ** denote significant differences at P<0.05 and P<0.01, respectively, determined through one-way ANOVA with subsequent Tukey’s multiple comparison test.

To explore sugar accumulation and the associated changes in the processing quality of potatoes during post-harvest storage under varying conditions, freshly harvested potatoes were stored at 4°C for intervals of 0, 1, 3, 5, 10, and 15 days, as well as at 20°C for 5, 10, and 15 days. We quantified sucrose, RS (glucose and fructose), and asparagine. As expected, glucose and fructose accrued significantly during cold storage, with a notable rise from day five at the lower temperature (**Figure 1A**). Conversely, at 20°C, the levels of RS remained constant and were significantly lower than in tubers kept at colder temperatures (**Figure 1A**). Intriguingly, sucrose levels dropped after five days, decreased further by day ten, and then increased on day fifteen (**Figure 1A**). This pattern might indicate the utilization of sucrose for RS formation, followed by a later re-synthesis of sucrose. Asparagine content was considerably higher during storage at ambient temperatures than cold storage (**Figure 1A**), hinting that CIS is primarily due to sugar accumulation rather than asparagine buildup.

Subsequently, we conducted a processing quality analysis of these tubers. Mirroring the changes in RS content, the color of potato chips processed after extended cold storage progressively darkened, whereas those stored at ambient temperature exhibited a lighter hue (**Figures 1B, C**). The alteration in color is further corroborated by colorimetric quantification using a chroma meter (**Figure 1D**). Aligning with the variations in RS content and processing color, results from acrylamide assays demonstrated that post-cold storage, the acrylamide content in fried chipping was significantly elevated compared to pre-storage levels and those stored at ambient temperature (**Figure 1E**).

### 3.2 Dynamic transcriptome profiling of tuber during storage and validation of RNA-seq results

**Figure 2:**
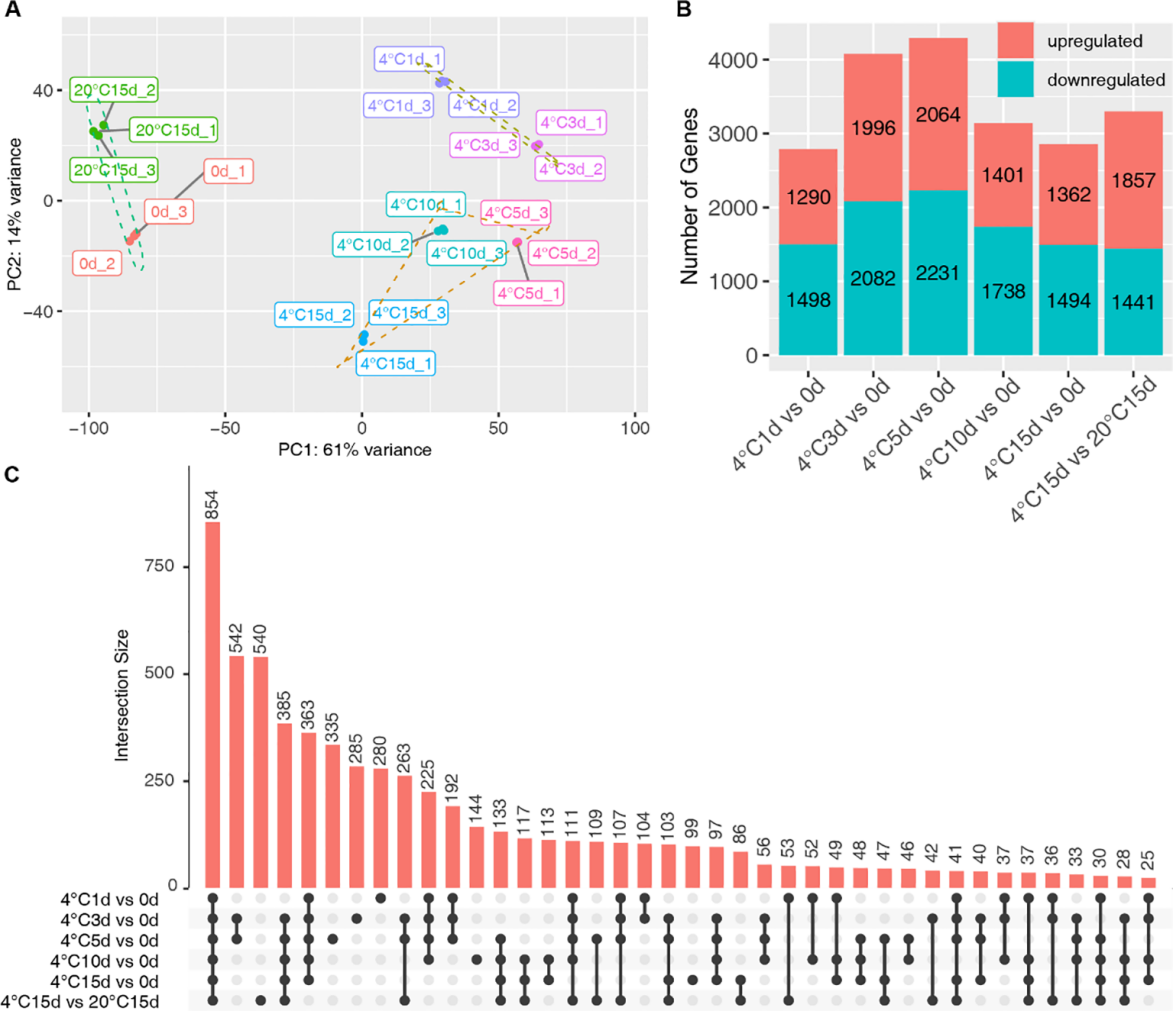
Transcriptomic analysis of potato tubers subjected to varied storage conditions. **(A)** Principal Component Analysis (PCA) of transcriptomic sequencing samples. **(B)** Comparative analysis of genes upregulated and downregulated across different storage durations, in contrast to 0 days of storage. **(C)** The Upset plot presents the DEGs across various storage periods. The upper section of the plot indicates the number of DEGs that intersect among the treatments, while the left-hand side chart enumerates the DEGs specific to each storage duration. Single black dots symbolize DEGs unique to one dataset. When DEGs appear in two or more datasets, this is represented by interconnected black dots. These connections show where the datasets intersect, and the black dots along the line identify the corresponding datasets involved.

To comprehensively explore the transcriptional dynamics of tubers during storage, we selected samples from 0, 1, 3, 5, 10, and 15 days of cold storage (at 4°C) and 15 days at 20°C for RNA-Seq analysis. The Principal Component Analysis (PCA) results revealed that samples from identical groups were closely clustered. Further PCA clustering indicated that the samples could be categorized into three distinct clusters: those stored for 0 days and those stored at 20°C for 15 days, those undergoing cold storage for 1 and 3 days, and those in cold storage for 5, 10, and 15 days (**Figure 2A**). The analysis of differentially expressed genes (DEGs) was conducted using a fold change threshold of ≥ 2 and an FDR (False Discovery Rate) of <0.01. The comparisons evaluated DEGs between samples stored at 4°C for 1, 3, 5, 10, and 15 days against the 0-day reference and between samples stored at 4°C and 20°C for 15 days. The contrast 4°C 5d *versus* 0d yielded the highest number of DEGs, while the comparison 4°C 1d *versus* 0d exhibited the lowest count of differential gene expression (**Figure 2B**). The upset analysis illustrated dynamic transcriptome profiling of the tuber during storage. Interestingly, the intersection of DEGs, signifying the core set consistently modulated across all comparisons, exhibited the largest overlap, comprising 854 genes (**Figure 2C**). This intersecting subset represents a shared transcriptional signature robustly altered in response to cold storage. Notably, the union of DEGs across all contrasts encompassed 6,585 non-redundant genes (**Data S1**), representing the collective transcriptomic response to the cold storage and durations.

To validate the RNA-seq results, 12 DEGs representing diverse functional categories were selected for qRT-PCR analysis (**Supplementary Figure S1**). These included genes involved in various metabolic and regulatory processes: starch and sugar metabolism (*VInv*, *BAM1*, *BAM3.1*); heat stress response (*Heat Shock Protein 22*, *HSP22*; *Heat Shock Transcription Factor C1*, *HSFC1*; *HSFA2*); flowering processes (*Early Flowering 4*, *ELF4*; *Agamous-Like 16*, *AGL16*); plant hormone regulation (*Abscisic Acid Deficient 1*, *ABA1*); chlorophyll biosynthesis (*Glutamyl-tRNA Reductase 1*, *HEMA1*); RNA splicing (*Splicing Factor 35*, *SC35*); and cell wall modification (*Pectin Methylesterase Inhibitor 4*, *PMEI4*). **Supplementary Figure S1** illustrates the alignment between the trends observed in RNA-seq and qRT-PCR data following post-harvest storage. This congruence reinforces the RNA-seq findings’ reliability and reflects the transcriptome changes associated with these processes.

### 3.3 Identification of DEGs with a similar temporal expression pattern

**Figure 3:**
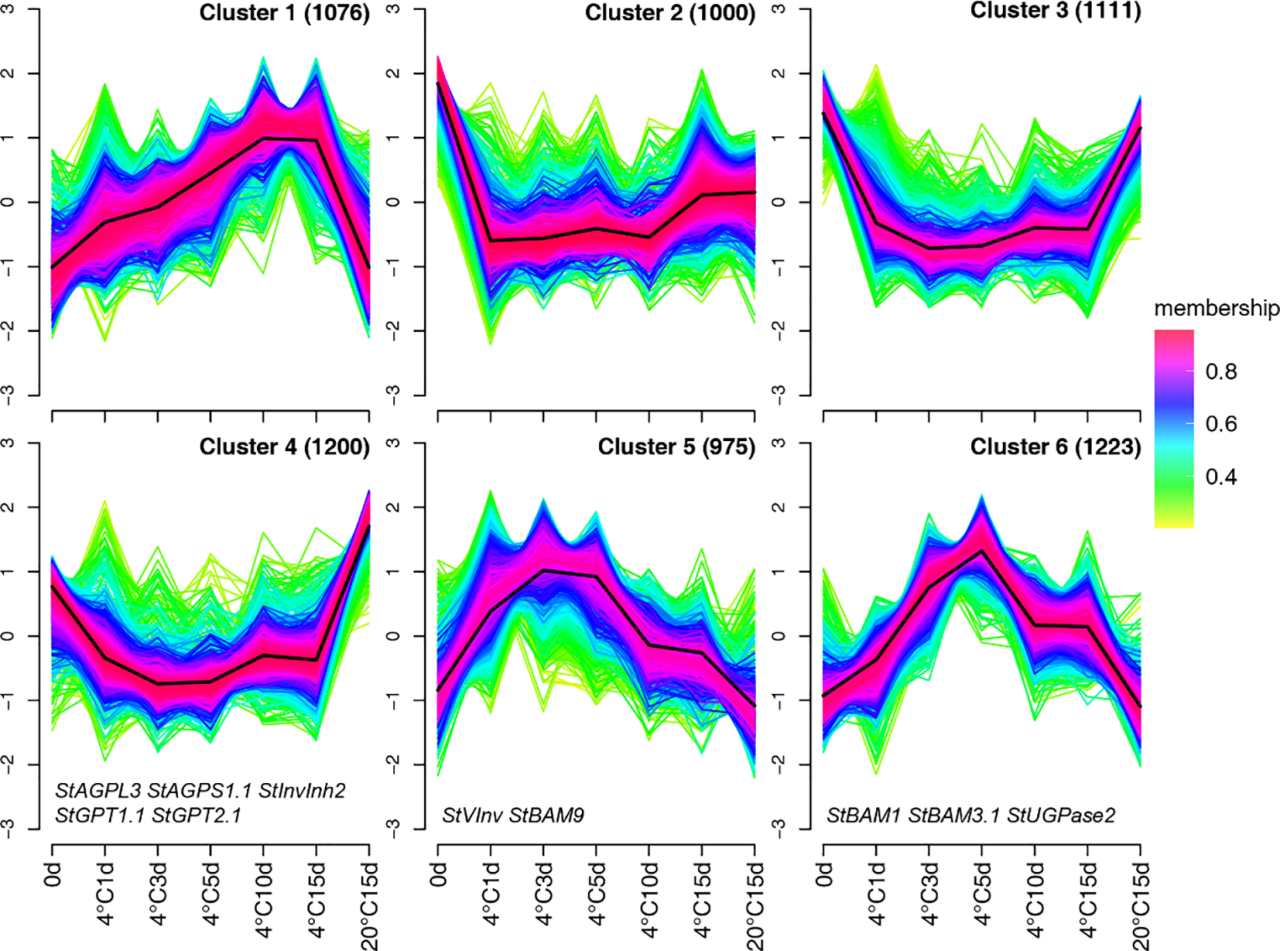
The Fuzzy c-means clustering method was employed to illustrate the major gene expression patterns observed under the seven treatment scenarios. This approach utilized the DEGs’ mean TPM values. The degree of a gene’s affiliation to a specific cluster is represented using a color gradient, where a deep red color (indicating an MS of 1) denotes a strong association with the cluster.

To explore the expression pattern of the DEGs during storage, we utilized fuzzy c-means (FCM) clustering to analyze the previously mentioned 6,585 DEGs. In this approach, each profile is assigned a membership degree across clusters. After an exhaustive assessment of various combinations of cluster sizes and fuzzification parameters, we found the optimal partitioning to be c = 6 and m = 1.75, resulting in 6 distinct clusters (**Figure 3**). Cluster 1, with 1,076 DEGs, exhibited the lowest expression at 0 days and 20°C storage for 15 days, but showed a notable increase in expression during later phases of cold storage at 10 and 15 days (**Figure 3**). Cluster 2, comprising 1,000 DEGs, exhibited initially subdued expression during cold storage, followed by a resurgence in expression at later stages, ultimately mirroring the levels observed at 15 days of 20°C storage, suggesting a potential non-involvement in the CIS. Cluster 3, including 1,111 DEGs, exhibited an immediate reduction in expression upon cold storage initiation and consistently maintained this level (**Figure 3**). Cluster 4 encompasses 1,200 DEGs exhibiting an expression pattern analogous to Cluster 3, with the distinction that their expression levels peaked after 15 days of storage at 20°C. This cluster is enriched with genes like *StAGPL3*, *StAGPS1.1*, *StGPT1.1*, and *StGPT2.1*, which are instrumental in starch synthesis (**Figure 3**). Clusters 5 and 6 show similar expression patterns induced during cold storage, with the notable difference being their temporal dynamics: Cluster 5 genes peak early in storage, while Cluster 6 peaks after 5 days of cold exposure. Crucial genes explicitly involved in CIS, such as *StVInv* and *StBAM9*, are represented in Cluster 5. Conversely, Cluster 6 encompasses notable CIS-related genes like *StBAM1* and *StUGPase2*. Regarding the striking enrichment of pivotal starch and sucrose metabolism genes within Clusters 4, 5, and 6, we postulate that these three clusters may constitute critical regulatory modules orchestrating the CIS process. Specifically, Cluster 4 contains genes downregulated during cold storage, including key starch synthesis genes, while Clusters 5 and 6 contain genes upregulated during cold storage, including crucial genes involved in starch degradation and sugar accumulation. This makes these clusters particularly relevant for understanding the molecular mechanisms of CIS. Consequently, we focused our subsequent analyses on these three gene clusters to elucidate their specific regulatory mechanisms and functional implications in CIS.

### 3.4 Functional enrichment analysis for DEGs in clusters 4, 5, and 6

**Figure 4:**
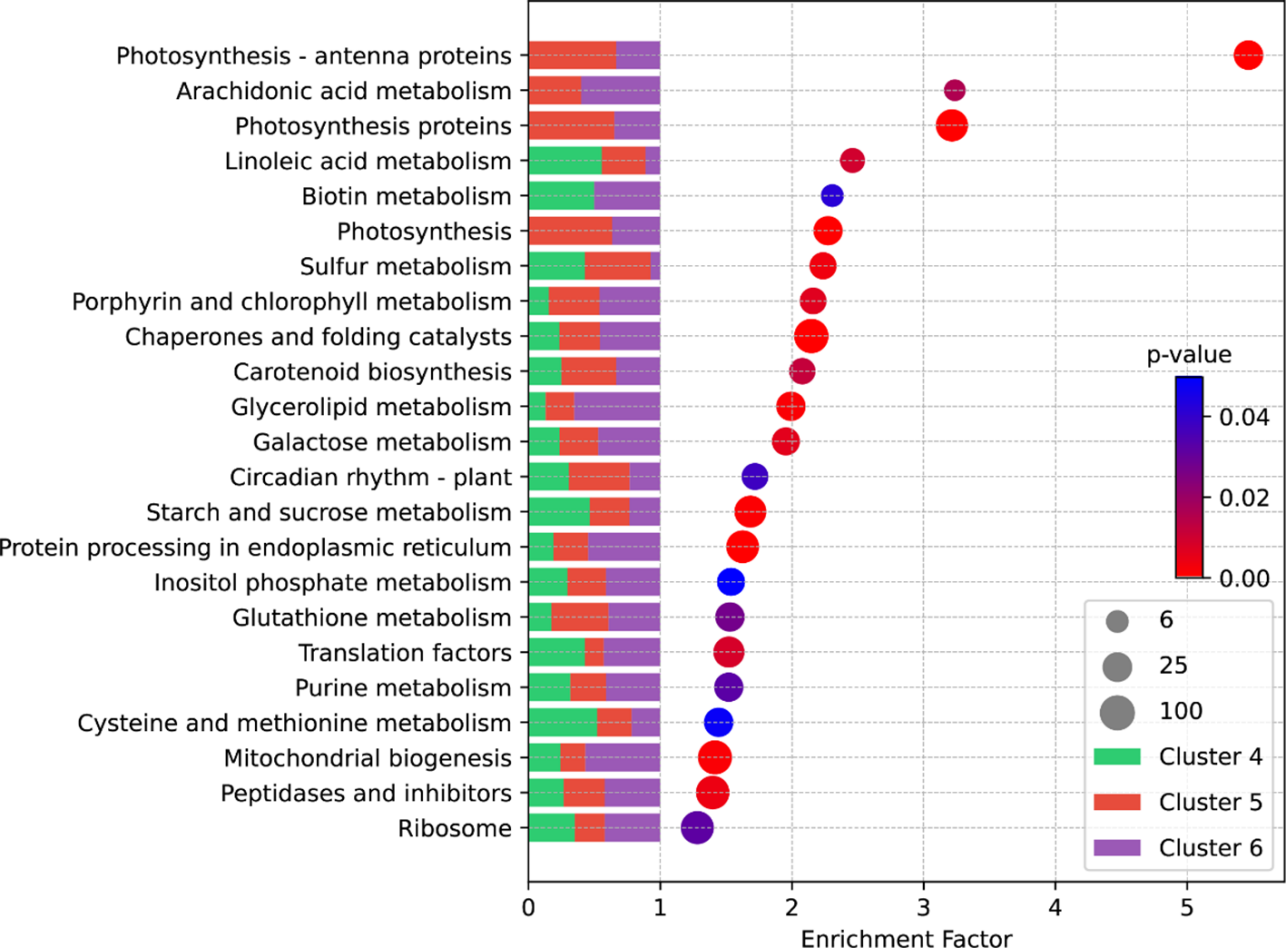
KEGG enrichment analysis of DEGs from Cluster 4, 5, and 6. Each circle denotes a specific metabolic pathway within the KEGG database. The circle’s size reflects the count of genes enriched within that pathway. The enrichment factor is determined using the formula: “Enrichment Factor = (Proportion of DEGs annotated within a specific term to the total DEGs)/(Proportion of all genes annotated within the same term to the total gene count).” The color reflects the p-value, with a smaller p-value indicating greater significance or reliability of the enrichment. The stacked bar graph on the left illustrates the proportion of DEGs from different clusters within the enriched pathway.

We selected DEGs from Clusters 4, 5, and 6 and subjected them to KEGG enrichment analyses. The KEGG analysis revealed that genes with a high enrichment factor were primarily associated with photosynthesis and its related pathways (**Figure 4**). Intriguingly, most genes associated with these pathways were derived from Clusters 5 and 6 (**Figure 4**), indicating that their expression is predominantly induced by cold storage. In addition, notable pathways included Arachidonic Acid metabolism, key molecules activating plant stress and defense signaling networks; carotenoid metabolism, which serves as a precursor route for ABA synthesis; circadian rhythm, an essential pathway for plants to adapt to environmental signals; and pathways related to starch and sugar metabolism such as Starch and Sucrose Metabolism, inositol phosphate metabolism, and Galactose Metabolism (**Figure 4**). The enrichment analysis suggests that low temperatures induce differential expression of a plethora of tuber genes, leading to alterations in the equilibrium of starch and sugar metabolism, ultimately resulting in CIS. Specifically, the imbalance in the Starch and Sucrose Metabolism pathway is the direct cause of sugar accumulation during cold storage. We placed particular emphasis on investigating the DEGs associated with this pathway.

### 3.5 Analysis of the differentially abundant metabolites after cold storage

**Figure 5:**
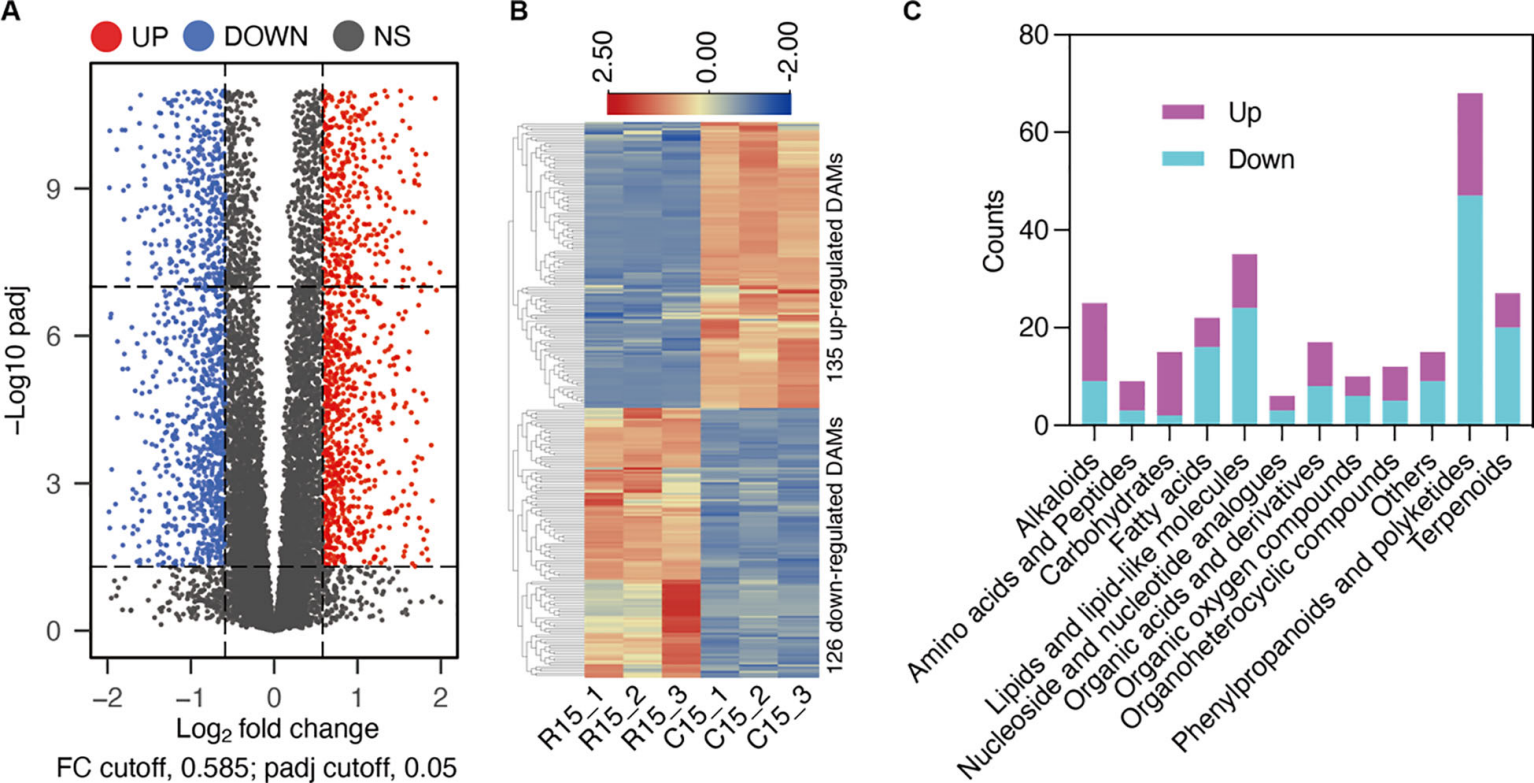
Metabolite profiles altered by cold storage conditions. **(A)** Comparative metabolomic analysis of room temperature (R15) and cold storage (C15) after 15 days. Volcano plots of DAMs in the investigated comparisons. **(B)** Heatmap illustrating the 135 up-regulated DAMs and 126 DAMs after cold storage. **(C)** Major classes of detected DAMs in C15/R15.

To elucidate the metabolic changes induced by cold storage, we employed metabolomics to determine the metabolomic differences between samples stored at 4°C for 15 days and those stored at 20°C for 15 days. The differential threshold was set to an FDR (False Discovery Rate) of less than 0.05 and a fold change of 1.5, corresponding to a log2FoldChange greater than or equal to 0.585. The results identified 261 Differentially Abundant Metabolites (DAMs), including 135 upregulated and 126 downregulated DAMs (**Figure 5**). Further analysis of these DAMs revealed that among the primary metabolites, most “Carbohydrates” and “Amino acids and peptides” exhibited increased levels after cold storage (**Figure 5**). In contrast “Fatty acids” and “Lipids and lipid-like molecules” showed a decrease in abundance. For secondary metabolites, the majority of “Phenylpropanoids and polyketides”, as well as “Terpenoids”, were downregulated, while most “Alkaloids” were upregulated during cold storage (**Figure 5**). These findings suggest cold storage induces significant alterations in the primary metabolic pathways.

The elevated levels of Carbohydrates and Amino acids and peptides indicate a potential shift in the energy metabolism and protein synthesis processes. Carbohydrates serve as a primary energy source for cellular functions, and their accumulation during cold storage may respond to reduced metabolic activity and energy demand under low-temperature conditions. Similarly, the increase in Amino acids and peptides suggests a possible upregulation of protein synthesis or a decrease in protein degradation rates. This could be a protective mechanism to maintain cellular integrity and function during cold stress.

On the other hand, the decrease in Fatty acids and Lipids and lipid-like molecules implies a downregulation of lipid metabolism during cold storage. Lipids are essential in membrane structure, energy storage, and signaling pathways. The reduction in their abundance may be attributed to cellular membranes’ decreased fluidity and permeability at low temperatures, leading to a reorganization of lipid composition to maintain membrane functionality. Additionally, the downregulation of lipid metabolism could result from the reduced energy requirements and the shift towards carbohydrate utilization as the primary energy source.

These metabolic changes provide valuable insights into the samples’ adaptive responses to low-temperature storage conditions. Further investigation of the specific metabolic pathways and their regulation mechanisms could contribute to a better understanding of the underlying molecular processes and potentially lead to developing strategies to enhance the quality and shelf life of the stored products.

### 3.6 Transcriptional and metabolic responses of starch-sugar interconversion pathway during cold storage

**Figure 6:**
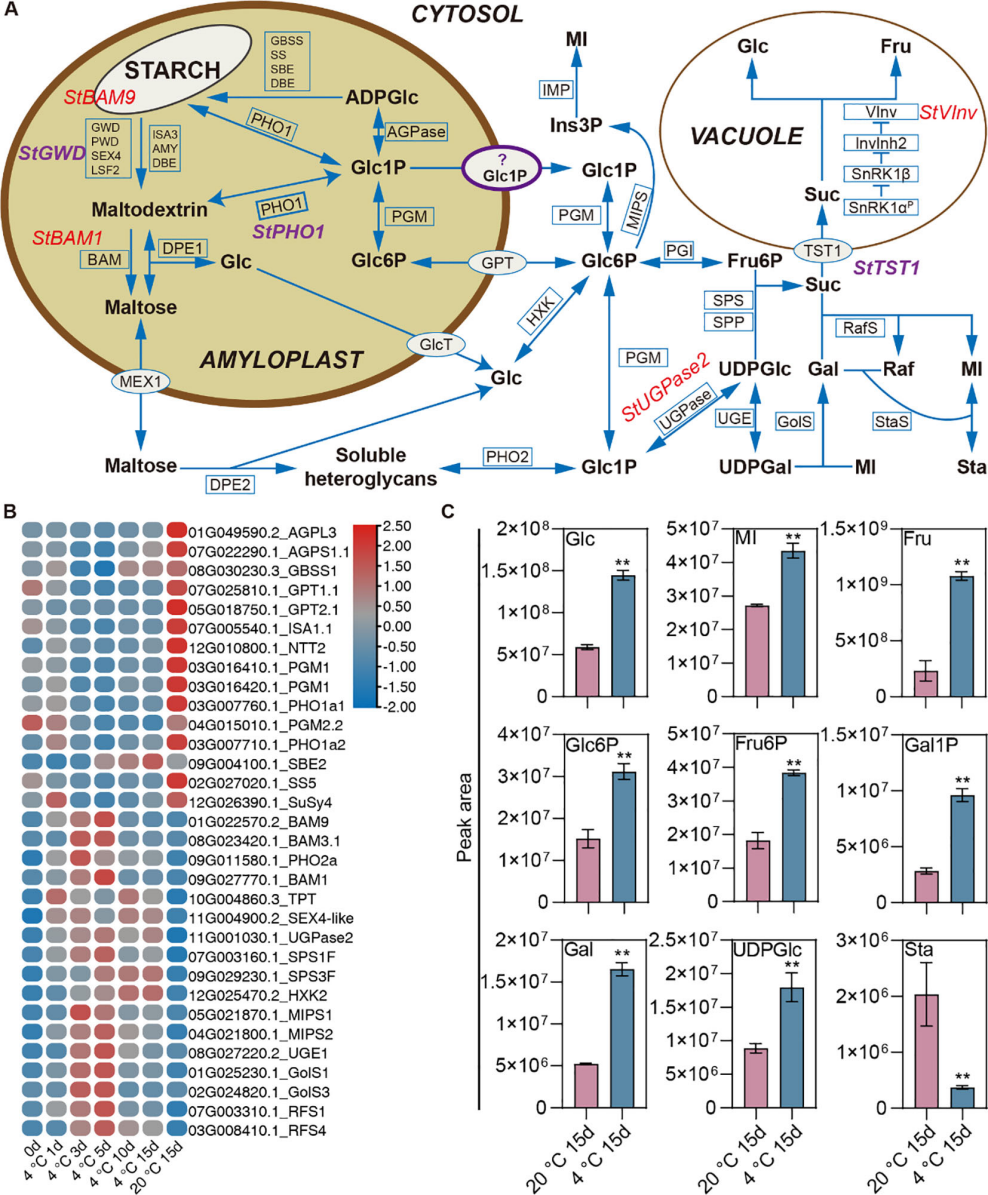
Transcriptional and Metabolic Analysis in Starch-Sugar Interconversion Pathway During Cold Storage. **(A)** Schematic representation of the starch-sugar metabolism pathway within the amyloplast and its associated transport and conversion processes in the cytosol and vacuole. Key enzymes and transporters involved in the breakdown and synthesis of starch and related carbohydrates are indicated. Enzymes and transporters include StBAM9, StGWD, StBAM1, and StPho1, among others, with specific metabolic products such as ADPGlc, Glc1P, Glc6P, and maltose highlighted. The pathway details the conversion of starch into glucose and other derivatives, the transport mechanisms across different cellular compartments, and the utilization of these metabolites in various biosynthetic routes. Notably, the role of key regulatory enzymes and the interaction between different sugar phosphates are emphasized. **(B)** Heatmap depicting the relative expression levels of various genes associated with starch-sugar metabolism across different conditions. The color gradient represents expression levels. **(C)** Bar graphs showing the DAMs related to sugars between tubers storage at cold and 20°C for 15 days. Each bar represents the mean peak area, with error bars indicating the standard deviation. ** denotes significant differences at P<0.01 determined through the student’s *t*-test.

We identified DEGs related to starch-sugar metabolism responding to cold storage based on GO and KEGG annotation information and integrating previous systematic analyses of potato starch-sugar metabolic pathways (**Figure 6A**) (Liu et al., 2022). Temporal gene expression analysis revealed heterogeneous kinetic patterns among these identified DEGs (**Figure 6B**). Overall, the expression of most starch synthesis-related genes was suppressed during cold storage, while most genes involved in starch degradation, sucrose resynthesis, and breakdown were induced under these conditions (**Figure 6B**). Specifically, genes encoding crucial enzymes or transport proteins in the starch synthesis pathway exhibited decreased expression during cold storage. These included genes encoding ADP glucose pyrophosphorylase (*AGPL3* and *AGPS1.1*), granule-bound starch synthase (*GBSS*) and soluble starch synthase and (*SS5*), glucose 6-phosphate/phosphate translocators (*GPT1.1* and *GPT2.1*), and phosphoglucomutase (*PGM1*) (**Figures 6A, B**). Notably, upregulated genes included *BAM1*, *BAM9*, and *BAM3.1*, which encode β-amylase isoforms; genes coding for UDP-glucose pyrophosphorylase 2 (*UGPase2*) and sucrose phosphate synthase (*SPS1F* and *SPS3F*), which are involved in sucrose biosynthesis; and the vacuolar invertase gene *VInv* (**Figures 6A, B**).

Cold storage induced significant changes in the profiles of the DAMs related to starch-sugar metabolism. Compared to ambient temperature conditions, cold storage resulted in significantly elevated levels of glucose, fructose, myo-inositol, and galactinol (**Figures 6A, C**). In addition, significant increases were also observed in the levels of phosphorylated sugars and sugar nucleotides, specifically glucose 6-phosphate, fructose 6-phosphate, galactose 1-phosphate, and UDP-glucose (**Figures 6A, C**). In conclusion, our comprehensive analysis of transcriptomic and metabolomic changes during cold storage in potato tubers reveals a coordinated reprogramming of carbohydrate metabolism.

### 3.7 Identification of the candidate transcription factors potentially involved in the starch-sugar pathway during CIS

**Figure 7:**
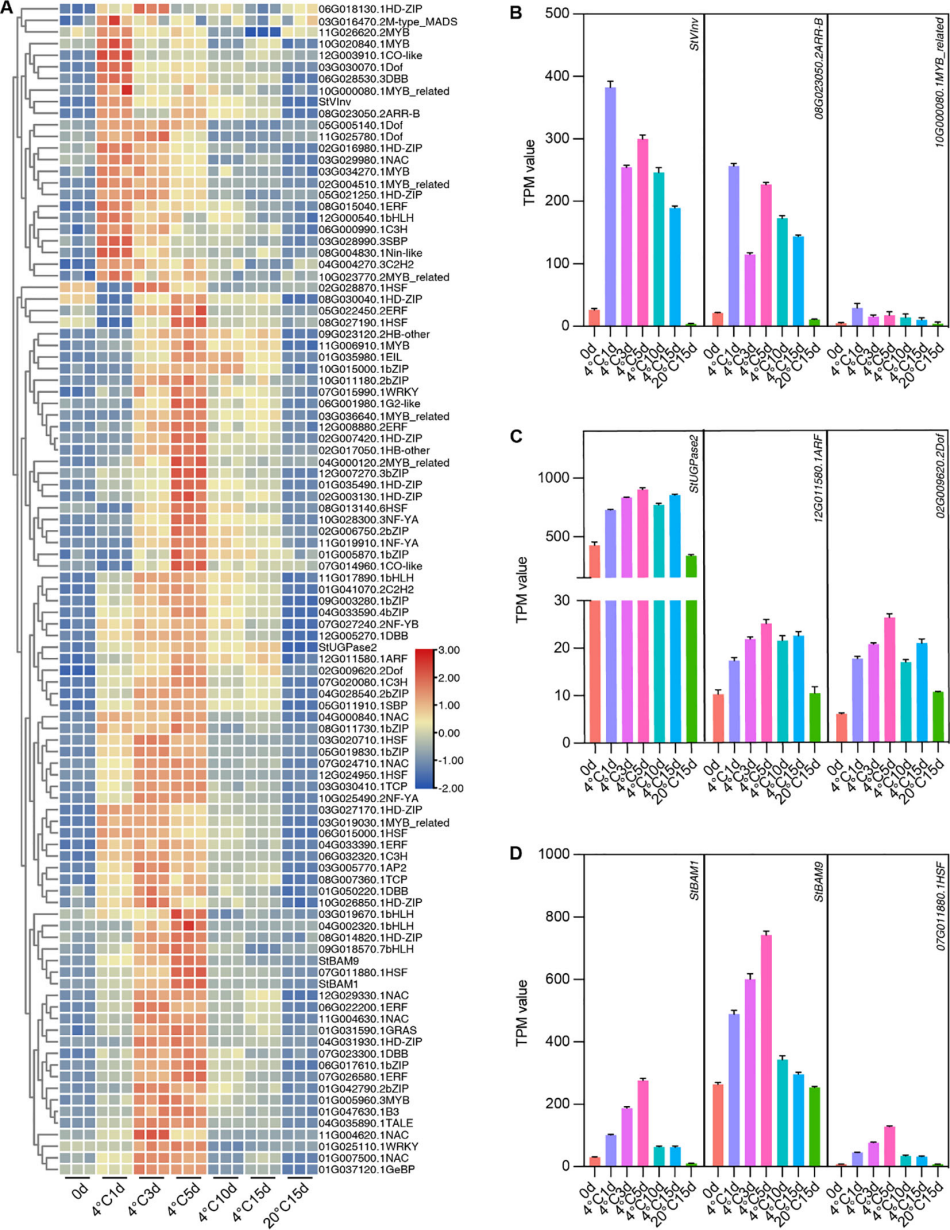
Screening for transcription factors co-expressed with CIS-related critical DEGs. **(A)** Heatmap Clustering Analysis of 97 Transcription Factors with Expression Patterns of *StVInv*, *StUGPase2*, *StBAM1*, and *StBAM9*. **(B-D)** Transcripts per million (TPM) values of *StVInv*, *StUGPase2*, *StBAM1*, and *StBAM9* with their co-expressed transcription factors. Data are presented as means ± SD (n=3).

Potential transcriptional regulators of these critical genes could be identified through co-expression analysis since transcriptional regulators often exhibit expression patterns similar to those of their targets. Among these genes, we mainly focused on *StUGPase2*, *StBAM1*, *StBAM9*, and *StVInv*, which have been reported to have defined functions in CIS (Bhaskar et al., 2010; Hou et al., 2017; Jaiswal et al., 2023). Within Cluster 5 and 6, 97 TF genes were identified. We performed a heatmap clustering analysis of 97 TFs with *StVInv*, *StUGPase*, *StBAM1*, and *StBAM9* expression patterns to determine the potential transcription factors regulating these critical genes. As illustrated in **Figure 7**, a MYB-related TF and an ARR-B TF were correlated with the expression of *StVInv*. Two TFs, including an ARF TF and a Dof TF, showed a similar expression pattern with *StUGPase*. *StBAM1*, *StBAM9*, and *StHSFA2* were clustered in the same subclass, indicating they exhibit highly consistent expression patterns (**Figure 7**). The co-expression analysis results suggest that these transcription factors may play a role in the transcriptional regulation of these critical genes within the starch and sugar metabolism pathway.

### 3.8 Functional characterization of StAPRR2 and StHSFA2

**Figure 8:**
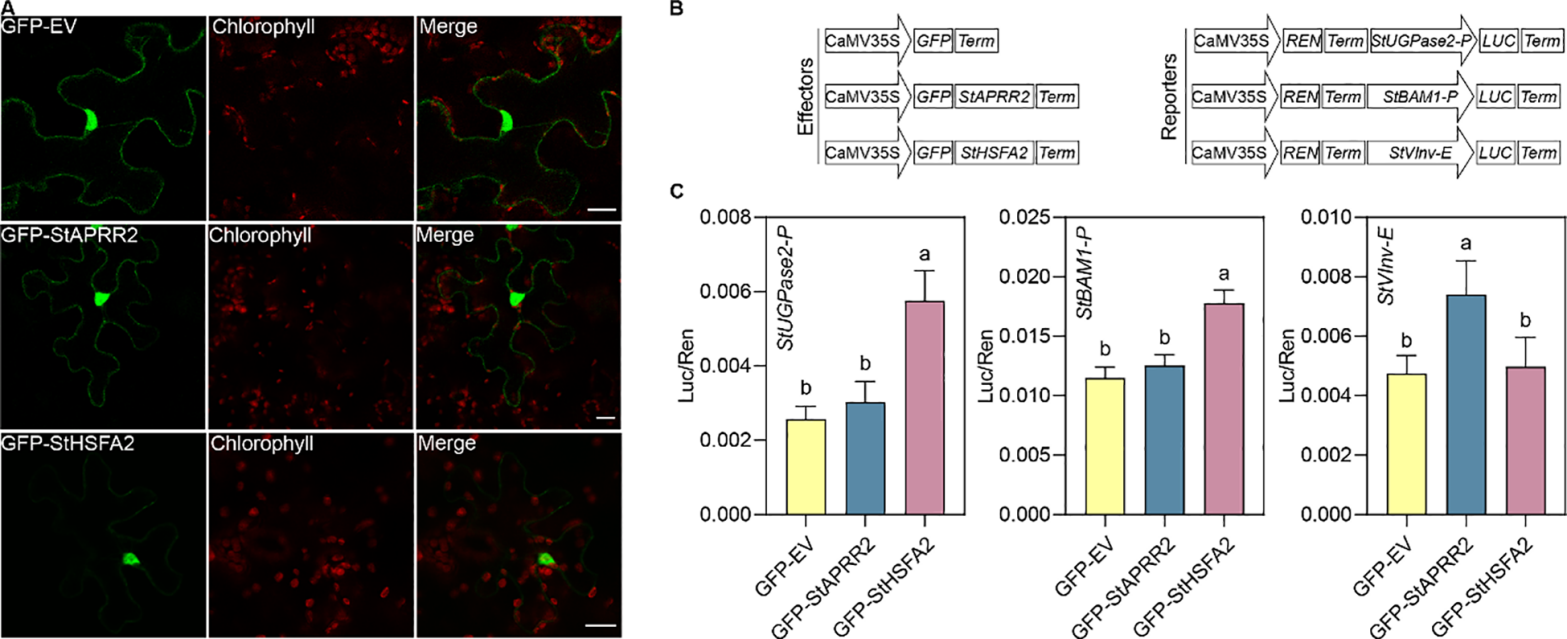
Subcellular localization and transactivation activity of StAPRR2 and StHSFA2. **(A)** Subcellular localization of GFP-StAPRR2 and GFP-StHSFA2 in *N. benthamiana* leaves. Scale bars, 20 μm. **(B)** Schematic representation of constructs used in the transient dual-luciferase reporter assays. **(C)** *A. tumefaciens* containing the reporter plasmids were transiently co-expressed with *A*. *tumefaciens* harboring effector plasmids in *N. benthamiana* leaves. Luciferase activity was assessed 3 days post-infiltration. Sequential measurements of firefly luciferase (LUC) and renilla luciferase (REN) were conducted, and the LUC/REN ratio was used to determine the final transcriptional activation activity. Data are presented as the mean ± SD (n = 6). Statistical significance is indicated by different letters, as determined by Tukey’s multiple comparisons test.

Based on the co-expression analysis, we selected StAPRR2 and StHSFA2 to test their ability to activate the transcription of reporter genes driven by the promoters of *StBAM1* and *StUGPase2* and *StVInv* enhancer. Confocal microscopy of GFP fusion proteins expressed in *N. benthamiana* leaves confirmed the nuclear localization of both transcription factors, consistent with their expected functions (**Figure 8A**). Dual-luciferase reporter assays (**Figures 8B, C**) demonstrated that StHSFA2 significantly activated the promoters of StUGPase2 and StBAM1, suggesting its involvement in regulating starch degradation and UDP-glucose production. Interestingly, StAPRR2 showed a specific and significant activation of the *StVInv* enhancer (**Figures 8B, C**), indicating its potential role in modulating invertase expression crucial for sucrose breakdown. These differential activation patterns suggest that StHSFA2 and StAPRR2 may play complementary roles in the complex regulation of carbohydrate metabolism under cold storage conditions. StHSFA2 appears to be a key regulator of starch degradation pathways, while StAPRR2 might specifically influence sucrose metabolism through invertase regulation.

## 4 Discussion

Our integrated time-course transcriptomic and metabolomic analysis provides novel insights into the regulatory mechanisms underlying cold-induced sweetening (CIS) in potato tubers. The results reveal a complex molecular network involving coordinated transcriptional regulation of starch-sugar metabolism genes and significant metabolic reprogramming during cold storage.

Our transcriptomic analysis identified 6,585 genes differentially expressed during cold storage, with 854 core genes consistently modulated across all time points, suggesting a robust and coordinated transcriptional response to cold. The clustering analysis revealed distinct temporal expression patterns that correlate with specific metabolic processes. Most notably, Clusters 4, 5, and 6 contained key genes involved in starch and sugar metabolism, with different expression kinetics reflecting their roles in the CIS process. The downregulation of starch synthesis genes (Cluster 4) coupled with the upregulation of starch degradation and sucrose metabolic genes (Clusters 5 and 6) creates a metabolic imbalance favoring sugar accumulation during cold storage.

Our metabolomic analysis revealed significant changes in primary and secondary metabolites during cold storage. The accumulation of sugars, sugar phosphates, and phosphorylated intermediates directly correlated with the transcriptional changes in carbohydrate metabolism genes. This metabolic shift was characterized by increased levels of glucose, fructose, UDP-glucose, and various phosphorylated sugars, which are key intermediates in the starch-sugar interconversion pathways. These findings align with previous studies identifying sucrose breakdown and reducing sugar accumulation as central to the CIS mechanism (Liu et al., 2021), but our time-course analysis further reveals the dynamic nature of these metabolic changes.

The observed fluctuations in sucrose levels throughout the storage period—initial decrease followed by later resurgence—suggest complex regulatory mechanisms controlling the balance between sucrose breakdown and re-synthesis. This pattern likely reflects the sequential activation of different enzymatic pathways during the progression of cold storage. The concurrent downregulation of lipid metabolism indicates a broader metabolic reprogramming in response to cold stress, potentially shifting energy utilization toward carbohydrate metabolism and supporting membrane reorganization to maintain cellular integrity at low temperatures (Lu et al., 2020; Venzhik et al., 2023).

A key finding of our study is the identification of specific transcription factors potentially regulating the starch-sugar interconversion during CIS. Our co-expression analysis revealed that StHSFA2 clustered with crucial starch degradation genes (*StBAM1*, *StBAM9*), while StAPRR2 showed expression patterns similar to the vacuolar invertase gene (*StVInv*). Importantly, functional validation through dual-luciferase reporter assays confirmed that StHSFA2 significantly activates the promoters of both *StBAM1* and *StUGPase2*, while StAPRR2 specifically activates the *StVInv* enhancer.

These findings suggest that StHSFA2 and StAPRR2 play distinct but complementary roles in regulating different aspects of the CIS process. StHSFA2 appears to primarily regulate starch degradation through *StBAM1* activation and sucrose synthesis via *StUGPase2*, while StAPRR2 specifically regulates sucrose hydrolysis through *StVInv* activation. This targeted regulation at different steps of the starch-sugar interconversion pathway presents a sophisticated control mechanism for CIS.

Interestingly, we also observed a significant induction of heat shock proteins (HSPs) during cold storage (**Supplementary Figure S3**). This seemingly paradoxical response reflects the broader role of HSPs as molecular chaperones that protect cellular proteins from various stresses, including cold (Hartl and Hayer-Hartl, 2002). The concurrent upregulation of StHSFA2, a heat shock transcription factor, with HSPs and key starch degradation genes suggests a dual regulatory function for StHSFA2 in coordinating both stress response and metabolic adaptation during cold storage. This dual function allows StHSFA2 to simultaneously activate genes involved in starch breakdown and induce HSPs to mitigate cellular stress, providing a comprehensive regulatory mechanism that balances sugar accumulation and cellular protection during CIS (Lin et al., 2019).

From a regulatory perspective, our identification of StHSFA2 and StAPRR2 as key transcription factors in the CIS process offers new insights into how potato tubers sense and respond to cold. Heat shock factors like StHSFA2 are known to respond to various stresses through direct sensing of cellular perturbations (Åkerfelt et al., 2010), suggesting that StHSFA2 may serve as a direct cold sensor that initiates the starch degradation cascade. Meanwhile, APRR proteins are components of the circadian clock machinery in plants, indicating that StAPRR2 may integrate cold signals with the internal timing mechanisms to regulate sucrose metabolism during extended cold storage.

The identification of these distinct but coordinated regulatory mechanisms advances our understanding of how potato tubers respond to cold storage and provides potential targets for genetic manipulation to mitigate CIS. Our finding that StHSFA2 activates both StBAM1 and StUGPase2 suggests that modifying this single transcription factor could simultaneously affect multiple steps in the starch degradation pathway, potentially offering a more effective approach to reducing sugar accumulation than targeting individual metabolic enzymes.

Although our study provides significant insights into the molecular mechanisms of CIS, several limitations should be acknowledged. First, our findings are based on a single potato variety (‘Netherlands No. 15’), and potato cultivars are known to vary considerably in their susceptibility to CIS. Therefore, the generalizability of these regulatory mechanisms across different potato genotypes requires further investigation. Second, while our functional validation confirms the ability of StHSFA2 and StAPRR2 to activate their respective target genes, comprehensive genetic studies involving gene editing or overexpression in potato tubers would provide more definitive evidence of their roles in regulating CIS *in vivo*.

Future research should expand this analysis to include multiple potato varieties with varying CIS susceptibility, which could reveal additional regulatory mechanisms and potential breeding targets. More extensive functional validation through genetic manipulation and analysis of natural variants could further elucidate the precise roles of StHSFA2 and StAPRR2 in CIS. Additionally, investigating the potential interplay between these transcription factors and other signaling pathways, such as plant hormone signaling and circadian regulation, could provide a more comprehensive understanding of the CIS process.

In conclusion, our integrated time-course analysis has revealed a coordinated transcriptional and metabolic network regulating CIS in potato tubers, with StHSFA2 and StAPRR2 serving as key regulatory nodes controlling different aspects of the starch-sugar interconversion pathway. These findings not only advance our fundamental understanding of cold adaptation in potato tubers but also identify specific molecular targets for developing CIS-resistant potato varieties through genetic engineering or marker-assisted breeding. Such improvements would significantly benefit the potato processing industry by reducing processing losses and improving the quality and safety of processed potato products.

## Data Availability

The data presented in the study are deposited in the Harvard Dataverse repository, and the mean TPM value of gene expression in potato tubers during storage can be obtained through the link https://doi.org/10.7910/DVN/L6QHKS.

## References

[B1] Åkerfelt M. Morimoto R. I. Sistonen L. (2010). Heat shock factors: integrators of cell stress, development and lifespan. Nat. Rev. Mol. Cell Biol. 11, 545–555. doi: 10.1038/nrm2938 20628411 PMC3402356

[B2] Bhaskar P. B. Wu L. Busse J. S. Whitty B. R. Hamernik A. J. Jansky S. H.. (2010). Suppression of the vacuolar invertase gene prevents cold-induced sweetening in potato. Plant Physiol. 154, 939–948. doi: 10.1104/pp.110.162545 20736383 PMC2948980

[B3] Chen C. Chen H. Zhang Y. Thomas H. R. Frank M. H. He Y.. (2020). TBtools: an integrative toolkit developed for interactive analyses of big biological data. Mol. Plant 13, 1194–1202. doi: 10.1016/j.molp.2020.06.009 32585190

[B4] Conway J. R. Lex A. Gehlenborg N. (2017). UpSetR: an R package for the visualization of intersecting sets and their properties. Bioinformatics 33, 2938–2940. doi: 10.1093/bioinformatics/btx364 28645171 PMC5870712

[B5] Hartl F. U. Hayer-Hartl M. (2002). Molecular chaperones in the cytosol: from nascent chain to folded protein. Science 295, 1852–1858. doi: 10.1126/science.1068408 11884745

[B6] Hou J. Zhang H. Liu J. Reid S. Liu T. Xu S.. (2017). Amylases StAmy23, StBAM1 and StBAM9 regulate cold-induced sweetening of potato tubers in distinct ways. J. Exp. Bot. 68, 2317–2331. doi: 10.1093/jxb/erx076 28369567 PMC5447890

[B7] Jaiswal S. Paul K. Raman K. V. Tyagi S. Saakre M. Tilgam J.. (2023). Amelioration of cold-induced sweetening in potato by RNAi mediated silencing of StUGPase encoding UDP-glucose pyrophosphorylase. Front. Plant Sci. 14, 1133029. doi: 10.3389/fpls.2023.1133029 36875591 PMC9981964

[B8] Kumar L. Futschik M. ,. E. (2007). Mfuzz: a software package for soft clustering of microarray data. Bioinformation 2, 5–7. doi: 10.6026/97320630002005 18084642 PMC2139991

[B9] Lin Y. Liu T. Liu J. Liu X. Ou Y. Zhang H.. (2015). Subtle regulation of potato acid invertase activity by a protein complex of invertase, invertase inhibitor, and SUCROSE NONFERMENTING1-RELATED PROTEIN KINASE. Plant Physiol. 168, 1807–1819. doi: 10.1104/pp.15.00664 26134163 PMC4528764

[B10] Lin Q. Xie Y. Guan W. Duan Y. Wang Z. Sun C. (2019). Combined transcriptomic and proteomic analysis of cold stress induced sugar accumulation and heat shock proteins expression during postharvest potato tuber storage. Food Chem. 297, 124991. doi: 10.1016/j.foodchem.2019.124991 31253316

[B11] Liu X. Chen L. Shi W. Xu X. Li Z. Liu T.. (2021). Comparative transcriptome reveals distinct starch-sugar interconversion patterns in potato genotypes contrasting for cold-induced sweetening capacity. Food Chem. 334, 127550. doi: 10.1016/j.foodchem.2020.127550 32693335

[B12] Liu T. Dong L. Wang E. Liu S. Cheng Y. Zhao J.. (2023a). StHAB1, a negative regulatory factor in abscisic acid signaling, plays crucial roles in potato drought tolerance and shoot branching. J. Exp. Bot. 74 (21), 6708–6721. doi: 10.1093/jxb/erad292 37479226

[B13] Liu T. Kawochar M. A. Begum S. Wang E. Zhou T. Jing S.. (2023b). Potato tonoplast sugar transporter 1 controls tuber sugar accumulation during postharvest cold storage. Hortic. Res. 10. doi: 10.1093/hr/uhad035 PMC10548405 37799627

[B14] Liu X. Lin Y. Liu J. Song B. Ou Y. Zhang H.. (2013). StInvInh2 as an inhibitor of StvacINV1 regulates the cold-induced sweetening of potato tubers by specifically capping vacuolar invertase activity. Plant Biotechnol. J. 11, 640–647. doi: 10.1111/pbi.2013.11.issue-5 23421503

[B15] Liu S. Liu T. Wang E. Cheng Y. Liu T. Chen G.. (2022). Dissecting the chloroplast proteome of the potato (Solanum tuberosum L.) and its comparison with the tuber amyloplast proteome. Plants 11, 1915. doi: 10.3390/plants11151915 35893618 PMC9332351

[B16] Liu X. Zhang C. Ou Y. Lin Y. Song B. Xie C.. (2011). Systematic analysis of potato acid invertase genes reveals that a cold-responsive member, StvacINV1, regulates cold-induced sweetening of tubers. Mol. Genet. Genomics 286, 109–118. doi: 10.1007/s00438-011-0632-1 21691778

[B17] Liu T. Zhou T. Lian M. Liu T. Hou J. Ijaz R.. (2019). Genome-wide identification and characterization of the AREB/ABF/ABI5 subfamily members from solanum tuberosum. Int. J. Mol. Sci. 20, 311. doi: 10.3390/ijms20020311 30646545 PMC6358972

[B18] Lorberth R. Ritte G. Willmitzer L. Kossmann J. (1998). Inhibition of a starch-granule–bound protein leads to modified starch and repression of cold sweetening. Nat. Biotechnol. 16, 473–477. doi: 10.1038/nbt0598-473 9592398

[B19] Love M. I. Huber W. Anders S. (2014). Moderated estimation of fold change and dispersion for RNA-seq data with DESeq2. Genome Biol. 15, 1–21. doi: 10.1186/s13059-014-0550-8 PMC4302049 25516281

[B20] Lu W.-J. Chen J.-Y. Shan W. Min T. Deng W. Chen Q.-F.. (2020). The membrane lipid metabolism in horticultural products suffering chilling injury. Food Qual Saf. 4, 9–14. doi: 10.1093/fqsafe/fyaa001

[B21] Lukasiewicz J. M. Van De Wiel C. C. M. Lotz L. A. P. Smulders M. J. M. (2024). Consumer transparency in the production chain for plant varieties produced using new genomic techniques. aBIOTECH 5, 239–246. doi: 10.1007/s42994-024-00142-y 38974855 PMC11224161

[B22] Maere S. Heymans K. Kuiper M. (2005). BiNGO: a Cytoscape plugin to assess overrepresentation of gene ontology categories in biological networks. Bioinformatics 21, 3448–3449. doi: 10.1093/bioinformatics/bti551 15972284

[B23] Mckenzie M. J. Chen R. K. Harris J. C. Ashworth M. J. Brummell D. A. (2013). Post-translational regulation of acid invertase activity by vacuolar invertase inhibitor affects resistance to cold-induced sweetening of potato tubers. Plant Cell Environ. 36, 176–185. doi: 10.1111/j.1365-3040.2012.02565.x 22734927

[B24] Patro R. Duggal G. Love M. I. Irizarry R. A. Kingsford C. (2017). Salmon provides fast and bias-aware quantification of transcript expression. Nat. Methods 14, 417–419. doi: 10.1038/nmeth.4197 28263959 PMC5600148

[B25] Shepherd L. Bradshaw J. Dale M. Mcnicol J. Pont S. Mottram D. S.. (2010). Variation in acrylamide producing potential in potato: segregation of the trait in a breeding population. Food Chem. 123, 568–573. doi: 10.1016/j.foodchem.2010.04.070

[B26] Shi W. Ma Q. Yin W. Liu T. Song Y. Chen Y.. (2022). The transcription factor StTINY3 enhances cold-induced sweetening resistance by coordinating starch resynthesis and sucrose hydrolysis in potato. J. Exp. Bot. 73, 4968–4980. doi: 10.1093/jxb/erac171 35511088

[B27] Shi W. Song Y. Liu T. Ma Q. Yin W. Shen Y.. (2021). StRAP2. 3, an ERF-VII transcription factor, directly activates StInvInh2 to enhance cold-induced sweetening resistance in potato. Hortic. Res. 8. doi: 10.1038/s41438-021-00522-1 PMC8012585 33790269

[B28] Sowokinos J. R. (2001). Biochemical and molecular control of cold-induced sweetening in potatoes. Am. J. Potato Res. 78, 221–236. doi: 10.1007/BF02883548

[B29] Venzhik Y. Deryabin A. Moshkov I. (2023). Adaptive strategy of plant cells during chilling: Aspect of ultrastructural reorganization. Plant Sci. 332, 111722. doi: 10.1016/j.plantsci.2023.111722 37120035

[B30] Wiberley-Bradford A. E. Busse J. S. Jiang J. Bethke P. C. (2014). Sugar metabolism, chip color, invertase activity, and gene expression during long-term cold storage of potato (Solanum tuberosum) tubers from wild-type and vacuolar invertase silencing lines of Katahdin. BMC Res. Notes 7, 1–10. doi: 10.1186/1756-0500-7-801 25399251 PMC4239387

[B31] Zhang H. Liu J. Hou J. Yao Y. Lin Y. Ou Y.. (2014). The potato amylase inhibitor gene SbAI regulates cold-induced sweetening in potato tubers by modulating amylase activity. Plant Biotechnol. J. 12, 984–993. doi: 10.1111/pbi.12221 24985879

[B32] Zhu X. Chen A. Butler N. M. Zeng Z. Xin H. Wang L.. (2024). Molecular dissection of an intronic enhancer governing cold-induced expression of the vacuolar invertase gene in potato. Plant Cell 36, 1985–1999. doi: 10.1093/plcell/koae050 38374801 PMC11062429

