# Supplementary material for: Deciphering the regulatory mechanisms of potato cold-induced sweetening via integrated time-course transcriptome and metabolome analysis
Source: Front Plant Sci. 2025 Apr 15;16:1551265. doi: 10.3389/fpls.2025.1551265 (PMC12037366; doi:10.3389/fpls.2025.1551265)
Supplement: Supplementary file 1 [file Table1.docx]

## Deciphering the regulatory mechanisms of potato cold-induced sweetening via integrated time-course transcriptome and metabolome analysis

Guangcan Cui^1^, Tingting Zhou^2, 3^, Zhongliang Liu^4^, Ting Wang^4^, Qingguo Wang^1*^, Tengfei Liu^1*^

^1^ College of Food Science and Engineering, Shandong Agricultural University, Tai’an 271018, Shandong, China

^2^ State Key Laboratory of Wheat Breeding, College of Agronomy, Shandong Agricultural University, Tai'an 271018, China

^3^ Department of Agronomy, Kansas State University, Manhattan, KS 66506, U.S.A.

^4^ Tai’an Academy of Agricultural Sciences, Tai’an 271000, China

^*^ Corresponding author:

E-mail addresses: wqgyyy@126.com (Q. Wang)

[hzauzsmj@gmail.com](mailto:hzauzsmj@gmail.com)(T. Liu)

**Table S1 Primers used in this research**

| Primers | Sequence(5’-3') | Related experiment |
| --- | --- | --- |
| q-ef1α-F | ATTGGAAACGGATATGCTCCA | q-RT-PCR |
| q-ef1α-R | TCCTTACCTGAACGCCTGTCA | q-RT-PCR |
| q-HSP22-F | ACGCCCCTTTTGGGACATAG | q-RT-PCR |
| q-HSP22-R | GGCGATTGATTCGACCCCTT | q-RT-PCR |
| q-HEMA1-F | TTCCACCTGTTGGTGCAAGT | q-RT-PCR |
| q-HEMA1-R | TTGTACACCCGTGCATCCTC | q-RT-PCR |
| q-ABA1-F | ACATGGCACCTGGGTTACAG | q-RT-PCR |
| q-ABA1-R | CCTTCCTTTCAGTAGTTTTTGGAGG | q-RT-PCR |
| q-HSFC1-F | CACGGCTCCACCAATGGATA | q-RT-PCR |
| q-HSFC1-R | CCGTAGTTCCTCCTCCTCCA | q-RT-PCR |
| q-vacINV1-F | GGCCACCCAGTACCATTCC | q-RT-PCR |
| q-vacINV1-R | GAATCGGGTTGATCCGGGAG | q-RT-PCR |
| q-BAM3.1-F | TGGAAATTGAGAAAAGAGAGAAGC | q-RT-PCR |
| q-BAM3.1-R | CAAGCTCGCATTCATCGCTC | q-RT-PCR |
| q-BAM1-F | GAAGGAAGGTGGTGGATGGG | q-RT-PCR |
| q-BAM1-R | CCTCGAATATCGCCTTGGCT | q-RT-PCR |
| q-HSFA2-F | TAACAGCAGCCCAGAGATGG | q-RT-PCR |
| q-HSFA2-R | GCACTCGGATGGGTTTGAGA | q-RT-PCR |
| q-ELF4-F | CAGGTGCAGTCTGTGTTGGA | q-RT-PCR |
| q-ELF4-R | ACCATGCTATCGTGCGTTCT | q-RT-PCR |
| q-AGL16-F | CAGAAGTCCCCTTGTTGCGA | q-RT-PCR |
| q-AGL16-R | CTGCGAATGCAGTTGTAGCC | q-RT-PCR |
| q-SC35-F | CATGGAAGAAGTGCCTCCCC | q-RT-PCR |
| q-SC35-R | ATCAGCATCAGAACGTGGCA | q-RT-PCR |
| q-PMEI4-F | ATCTCCCAAGCACAAGGCAA | q-RT-PCR |
| q-PMEI4-R | TAGCGATGTCAAGGGCAGTG | q-RT-PCR |
| HSFA2-LicF | ATTACGCCGAGGTCATGGAAGAGGAAACAATGAG | Subcellular localization |
| HSFA2-LicR | TAGGGAAGAGGTTAAGGCATACACTTGCACT | Subcellular localization |
| APRR-B-LicF | ATTACGCCGAGGTCATGATTTGCATTGAGAATGA | Subcellular localization |
| APRR-B-LicR | TAGGGAAGAGGTCATCTTCGATAACGGGAGC | Subcellular localization |
| UGPase2-jF | atagggcgaattgggtaccttgtaccgtataagactcacttcc | LUC |
| UGPase2-jR | ttttggcgtcttccatgggagaaagttctcgaggagca | LUC |
| INV1-intronjF | atagggcgaattgggtaccCACGACAAGTCAAATAGAGTAGGA | LUC |
| INV1-intronjR | ttttggcgtcttccatggGGCACAAGATAACCTACTCC | LUC |


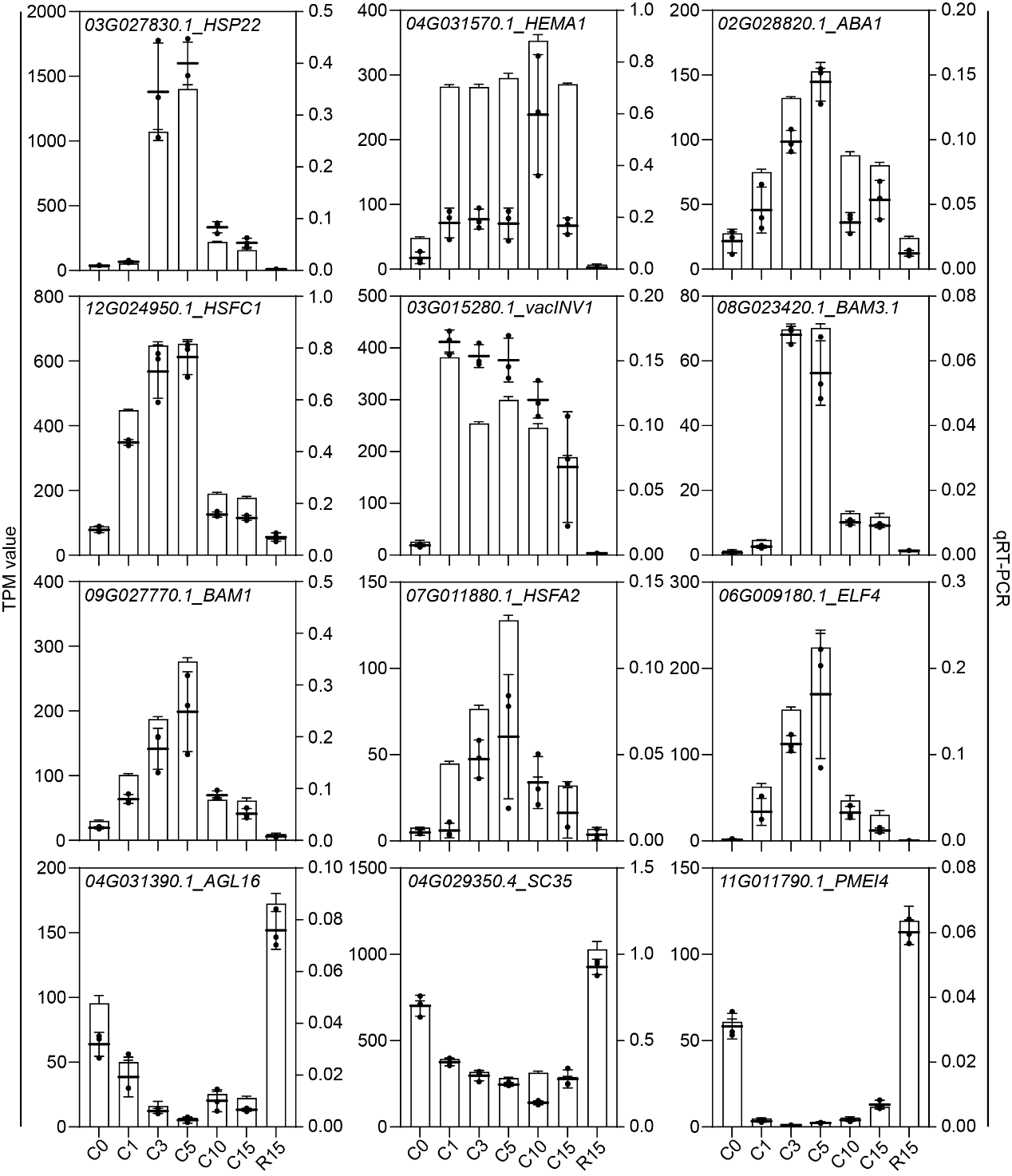


**Fig. S1** Quantitative RT-PCR validation of 12 selected DEGs identified by RNA-seq. C0 to R15 indicate the days stored under cold (C) or room (R) temperature conditions, respectively. The histograms depict RNA-seq results, as the Transcripts Per Kilobase Million (TPM) value indicates. The black dots represent qRT-PCR results. Values are shown as the mean ± SD (n = 3).


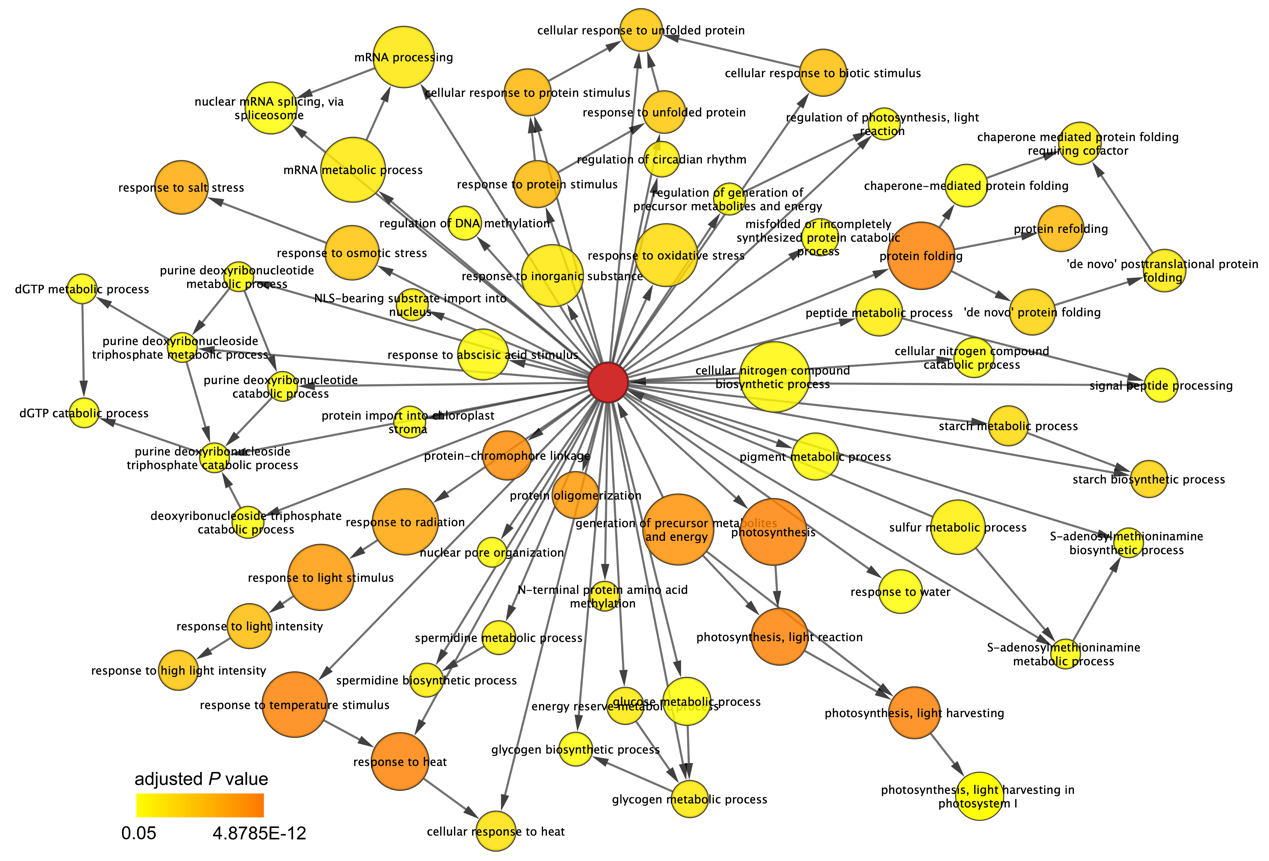


**Fig. S2** GO enrichment analysis of DEGs from Cluster 4, 5, and 6.


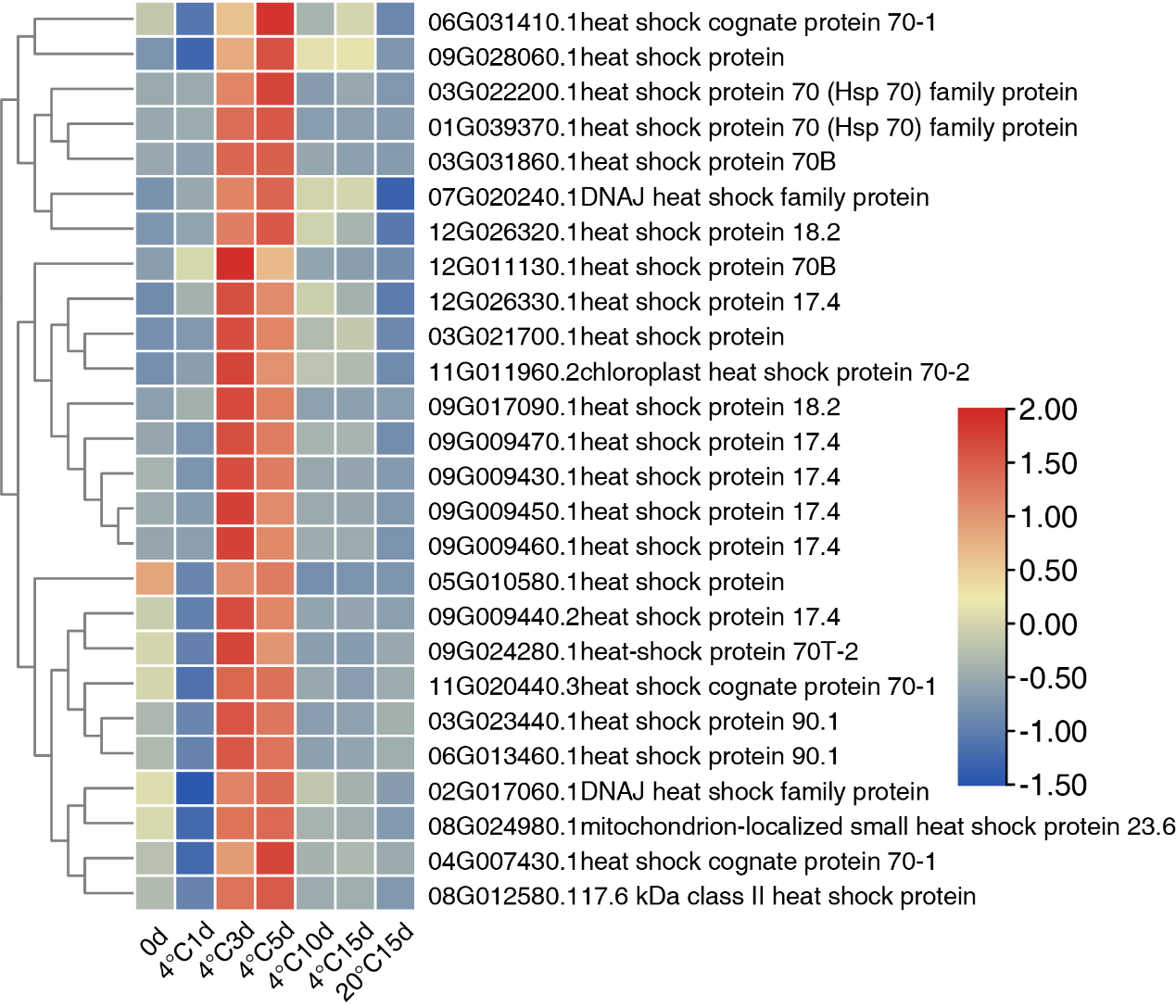


**Fig. S3** Heatmap Clustering Analysis of HSPs in Clusters 5 and 6.
